# Joint Selection for Growth and Leaf Color in Superior Trees of *Sapium discolor* in Fujian Province, China

**DOI:** 10.3390/plants15030452

**Published:** 2026-02-01

**Authors:** Yanghui Fang, Xuemei Wang, Liang Fang, Jie Guo, Wenping Chen, Wei Wu, Tong Wang, Zhixian Luo, Xun Lin, Daiquan Ye, Xiaochou Chen, Shunde Su

**Affiliations:** 1 College of Forestry and Grassland, Nanjing Forestry University, Nanjing 210037, China; 2 Fujian Provincial Forestry Science and Technology Promotion General Station, Fuzhou 350003, China; 3 Fuzhou Botanical Garden, Fuzhou 350012, China; 4 State Key Laboratory of Agricultural and Forestry Biosecurity, Fujian Agriculture and Forestry University, Fuzhou 350002, China; 5 College of Forestry, Fujian Agriculture and Forestry University, Fuzhou 350002, China; 6 Fujian Nanjing Yongfeng State-Owned Forest Farm, Nanjing 363602, China; 7 Fujian Jiangle State-Owned Forest Farm, Sanming 353300, China; 8 Fujian Yangkou State-Owned Forest Farm, Nanping 353211, China; 9 Fujian Academy of Forestry Sciences, Fuzhou 350012, China; ssdforest@163.com

**Keywords:** *Sapium discolor*, fast growth, red leaves, family, clonal propagation parent, genetic gain, genotypic value

## Abstract

*Sapium discolor* is a valuable native species in southern China, valued for its rapid growth and vibrant foliage, and widely used in ecological restoration and landscaping. To identify superior propagation materials with fast growth and red leaves, regional open-pollinated progeny trials of 10 elite trees were established in Nanping, Sanming, and Zhangzhou (Fujian Province) in 2015. Growth (tree height and diameter) was monitored from 2015 to 2023, and leaf color (the proportion of red in leaf color) was assessed in 2024. The species showed early fast growth, with mean tree height and diameter at breast height (DBH) reaching 7.98 m and 9.99 cm at six years, then slowing. Family-level phenotypic variation was limited. ANOVA revealed highly significant differences among families for growth traits from 2016 onward and for leaf color in 2024. Broad-sense heritability was moderate for 2023 tree height (0.3839), DBH (0.1879), and 2024 leaf color (0.2102), with low narrow-sense heritability, indicating non-additive genetic effects. Clonal selection based on genotypic values achieved notable genetic gains, especially for growth. One superior clone combined improvements in height (13.1%), diameter (10.1%), and red coloration (8.3%). These results highlight the value of clonal selection and the need to consider genotype × environment interactions in breeding programs.

## 1. Introduction

*Sapium discolor* (Champ. ex Benth.) Muell. Arg. is an important native tree species of the Euphorbiaceae family, valued for its ecological restoration, ornamental, and medicinal uses [1,2]. With the ongoing emphasis on ecological civilization and the use of indigenous species in China, *S. discolor* has become increasingly prominent in southern afforestation and urban landscaping, owing to its striking autumn foliage coloration and strong environmental adaptability [3,4].

Growth rate and leaf color are key traits that affect both the ecological and ornamental value of trees, and are thus central targets in forestry and horticultural breeding programs [5,6,7,8,9]. For ornamental trees such as *S. discolor*, the pigmentation and genetic variation in leaf color are influenced by both environmental and genetic factors [7,8,9]. Leaf coloration is primarily regulated by the accumulation of anthocyanins, carotenoids, and chlorophylls, which respond dynamically to light, temperature, and soil conditions [7,10,11]. Previous studies have shown that while leaf color traits exhibit a certain degree of heritability, non-additive genetic effects play a major role in pigment accumulation [11,12]. Therefore, genetic improvement of ornamental trees with colored foliage requires a multi-trait selection approach, combining growth and leaf color, through progeny tests, heritability estimation, and clonal selection to identify genotypes with rapid growth and desirable ornamental features [7,11].

Vigorous early growth is also crucial for the adaptation and large-scale promotion of plantation species. Progeny tests of various tree species have demonstrated that families with superior early growth often maintain their advantages at later stages [13,14,15]. The use of genetic parameters and heritability estimates can help determine optimal selection ages and strategies, thereby improving breeding efficiency [13]. For colored-leaf species, it is also important to consider the impact of geographic variation on leaf color expression, and to leverage regional differences for material integration and superior tree selection [10].

To date, studies focusing on the combined selection of superior growth and ornamental traits in *S. discolor* are still limited. Building on conventional tree breeding methodologies, the present study utilized open-pollinated progeny tests to jointly analyze the genetic variation and inheritance of growth and leaf color traits in *S. discolor*. The aim of this study was to identify superior clonal candidates combing rapid growth and attractive autumnal leaf coloration in Fujian Province, China, a region representing part of the natural distribution range of *S. discolor*. This study is expected to provide a theoretical and technical basis for genetic improvement, clonal selection, and high-quality afforestation of *S. discolor*, and to offer insights into multi-trait improvement strategies for ornamental tree breeding.

## 2. Materials and Methods

### 2.1. Site Description

#### 2.1.1. Site 1: Shunchang County, Nanping City, Fujian Province

The first experimental site was located at Datiekeng Work Area (Plot No.: 040-32-010), Yangkou State-owned Forest Farm, Shunchang County, Nanping City, Fujian Province, China. The site lies at 26°47′ N, 117°55′ E, with an elevation ranging from 50 to 150 m. The area is characterized by low hilly terrain, situated on the lower-to-mid-slopes with a southwesterly aspect and a slope gradient of 25°. The soil is classified as deep red soil (site class II). The region experiences a subtropical climate, with an average annual temperature of 18.5 °C, a maximum of 40.3 °C, and a minimum of −6.8 °C. Average annual precipitation is 1880 mm, with an average frost-free period of 280 days and a mean relative humidity of 82%.

#### 2.1.2. Site 2: Jianle County, Sanming City, Fujian Province

This site was established in the Mingtoushan Work Area (Plot No.: 061-13-090), Jiangle State-owned Forest Farm, Jiangle County, Sanming City, Fujian Province, China (26°43′ N, 117°27′ E; elevation 220–410 m). The site is located on the southeastern foothills of the Wuyi Mountains, in the middle and lower reaches of the Jinxi River, featuring mountainous and hilly landforms. The slope faces northeast with a gradient of 24°. The soil is deep red soil (site class II). The region has a mid-subtropical monsoon climate, with an average annual temperature of 18.7 °C (maximum 40.2 °C, minimum −6.9 °C), a mean annual precipitation of 1676 mm, a frost-free period of 299 days, and a mean relative humidity of 82%.

#### 2.1.3. Site 3: Nanjing County, Zhangzhou City, Fujian Province

This site was set up in Yongfeng State-owned Forest Farm (Plot No.: 002-21-020), Nanjing County, Zhangzhou City, Fujian Province, China. The site is located at 24°40′ N, 117°29′ E, at elevations of 420–520 m, on the lower- to mid-slopes with a northwestern aspect and a slope of 25°. The soil is deep red soil (site class II). The area has a southern subtropical, warm, and humid climate, with an average annual temperature of 21 °C, annual precipitation of 1700 mm, a 320-day frost-free period, and a mean relative humidity of 80%. The plantation was established in March 2015.

### 2.2. Plant Materials

In 2012, field surveys were conducted in Jiujiang County (Jiangxi Province), as well as Nanping and Zhangzhou (Fujian Province), to assess the growth environment and distribution of *S. discolor*. Ten superior trees were selected based on comprehensive evaluation of tree height, diameter at breast height (DBH), clear bole height, stem straightness, crown fullness, and natural pruning ability.

Open-pollinated progenies from these ten plus trees were used as experimental families. The family codes and superior tree characteristics are shown in Table 1. Seeds of the ten families were collected in mid-December 2012, subjected to seed quality testing, and stored in a cool, dry environment. In February 2013, seeds were pre-germinated, and after germination, seedlings were transplanted into a light substrate consisting of peat, coconut coir, and perlite (3:2:1, *v*/*v*/*v*), supplemented with 15 g·L^−1^ organic fertilizer. Uniform nursery management practices were implemented. The nursery was located at the Seedling Experimental Base of the Fujian Forestry Science and Technology Experimental Center (117°19.4035′ E, 24°30.7060′ N; elevation 91 m).

### 2.3. Experimental Design and Methods

A randomized complete block design (RCBD) was adopted, with 10 treatments (open-pollinated families derived from plus-tree progenies), 10 replicates (plots), and each plot consisting of 8 plants in double rows. Thus, each family was represented by 10 plots (one per replicate), totaling up to 80 trees per family and 800 trees in total (under full survival). The nursery was prepared with strip plowing (strip width: 0.8 m), and the planting spacing was 2.0 m × 2.0 m. Planting holes were 50 cm × 40 cm × 30 cm. The plantation was established in March 2015. During the first three years after planting, block weeding and tending were conducted in June–July and September–October each year.

Tree height and ground diameter were measured in December 2015 and December 2016, while tree height and DBH were measured in January 2021 and April 2024. In December 2024, leaf color was surveyed using DJI drone (unmanned aerial vehicle, UAV) RGB orthoimages (Mavic 3, DJ-Innovations, Shenzhen, China). UAV flights were conducted in early December (10:30–11:00) under sunny conditions when >75% of leaf color change had occurred. Images were acquired using nadir (vertical) imaging at an above-ground level (AGL) of 30 m and a flight speed of 3 m/s, with the camera set to automatically capture one image every 1.5 s. Image processing and orthomosaic (orthophoto) generation were performed using DJI Terra (DJ-Innovations, Shenzhen, China), and the mosaicking output was a true orthomosaic; subsequent leaf color analyses were conducted based on this orthomosaic. For individual-tree crown sampling, a per-tree region was delineated as a circle with a radius of 0.5 m centered on the crown center, and RGB values within this circle were extracted as representative values for each tree. The red color ratio of leaves was quantified as 2024R = Red/(Red + Green + Blue), where Red, Green, and Blue represent the respective color values of each tree, estimated using ArcGIS software (ArcGIS 10.8, Environmental Systems Research Institute, Redlands, CA, USA).

The linear model for variance analysis is as follows:

(1) $$Y_{ijkl}=\bar{x}+S_{i}+B_{j}+F_{k}+{\left(S\times F\right)}_{ik}+{\left(S\times B\times F\right)}_{ijk}+E_{ijkl}$$

where *Y_ijkl_* is the observation value of the *l*th plant in the *k*th family, *j*th block, *i*th site; $\bar{x}$ is the overall mean; *S*_i_ is the effect of the *i*th site; *B*_j_ is the effect of the *j*th block; *F*_k_ is the effect of the *k*th family; (*S* × *F*)*_ik_* is the interaction between site and family; (*S* × *B* × *F*)*_ijk_* is the interaction among site, block, and family; *E_ijk_* is the random error. $\bar{x}$, *S_i_*, *B_j_* are fixed effects, *F_k_*, *(S × F)_ik_*, *(S × B × F)_ijk_*, *E_ijkl_* are random effects.

Estimation formulas for genetic parameters are as follows [13]:

(2) $$\text{Broad-sense heritability:}\;h_{f}^{2}=\delta_{f}^{2}/\left(\delta_{e}^{2}/NBS+\delta_{fbs}^{2}/BS+\delta_{fs}^{2}/S+\delta_{f}^{2}\right)$$

(3) $$\text{Narrow-sense heritability:}\;h_{i}^{2}=4\delta_{f}^{2}/\left(\delta_{e}^{2}+\delta_{fs}^{2}+\delta_{fbs}^{2}+\delta_{f}^{2}\right)$$

(4) $$\text{Genotypic value:}\;G_{i}=h_{f}^{2}(x-\bar{x})$$

(5) $$\text{Breeding value:}\;G_{a}=h_{i}^{2}(x-\bar{x})$$

(6) $$\text{Clonal genetic gain:}\;\Delta G=\bar{G_{i}}/\bar{x}\times 100\%$$

where $\delta_{f}^{2}$ is the family variance component; $\delta_{fs}^{2}$ is the site × family interaction variance; $\delta_{fbs}^{2}$ is the site × block × family interaction variance; $\delta_{e}^{2}$ is the environmental variance; S is the number of sites; B is the number of replicates; N is the number of individuals per plot; $\bar{G_{i}}$ is the mean genotypic value of selected individuals. Variance components were estimated using the maximum likelihood method in IBM SPSS Statistics 19.0. The ratio of narrow-sense to broad-sense heritability (I = $h_{i}^{2}$⁄$h_{f}^{2}$) was used to measure the proportion of additive to total genetic variance. A lower I value indicates less additive and more non-additive genetic effects, favoring the selection of superior clones via clonal propagation; a higher value indicates the opposite [13].

## 3. Results

### 3.1. Phenotypic Variation Analysis

The minimum, maximum, mean, standard deviation, and coefficients of variation for average tree height, ground diameter, diameter at breast height (DBH), and the proportion of red in leaf color (2024) of the *S. discolor* progeny tests across three sites and over four years are presented in Table 2. The coefficients of variation among families for each trait and year was relatively low, ranging from 1.0% to 4.2%. As shown in Table 2, *S. discolor* exhibited rapid early growth, with average tree height and DBH reaching 7.98 m and 9.99 cm, respectively, six years after establishment, followed by a substantial slowdown in subsequent growth. This trend provides a solid basis for early selection.

### 3.2. Genetic Variation Analysis

Analysis of variance (ANOVA) for tree height, ground diameter, DBH, and red ratio in leaf color (Table 3) showed that differences among families for tree height in 2015 were not significant but became significant or highly significant from 2016 onwards. For ground diameter, differences among families in 2015 and 2016, and for DBH in 2020 were not significant, while a highly significant difference was observed for DBH in 2023. The proportion of red in leaf color in 2024 showed significant differences among families and highly significant differences among sites.

Genetic parameter estimates (Table 4) indicated that the genetic coefficients of variation among families for tree height in 2020 and 2023, DBH in 2023, and red ratio in leaf color in 2024 were 0.6%, 1.3%, 0.9%, and 0.5%, respectively, suggesting limited genetic differentiation among families. The broad-sense heritabilities for tree height and DBH in 2023 and red ratio in leaf color in 2024 were relatively low, at 0.3839, 0.1879, and 0.2102, respectively, while the narrow-sense heritabilities were all below 0.02. The ratio of narrow-sense to broad-sense heritability was less than 0.0738 for all three traits, indicating that while these traits are under some degree of genetic control, non-additive genetic effects are predominant. Therefore, clonal selection via vegetative propagation of superior individuals is recommended to maximize genetic gain, rather than selection of families or individuals for sexual breeding.

### 3.3. Selection of Clonal Candidate Trees

Using a 10% selection intensity, superior clones were identified based on genotypic values (Table 5). For tree height and DBH traits in 2023, candidate clones were all from Plus Tree No. 10, while for the red ratio in leaf color in 2024, Plus Tree No. 4 was selected. There was no overlap of plus trees with simultaneous improvement in both growth and leaf color traits; only Plus Tree No. 8 met both criteria.

Based on the 3σ principle of normal distribution and a 0.5% selection intensity, five clonal candidates were identified for each trait according to genotypic values (Table 6, Table 7 and Table 8). For tree height in 2023, the selected clones had average tree height, DBH, and red ratio values of 15.32 m, 18.02 cm, and 0.3558, with genetic gains of 25.2%, 10.8%, and 1.1%, respectively. For DBH in 2023, two clones overlapped with those selected by tree height, with average tree height, DBH, and red ratio of 13.22 m, 21.60 cm, and 0.3397, and genetic gains of 16.5%, 16.7%, and 0.1%, respectively. For the red ratio in leaf color in 2024, the selected clones showed mean tree height, DBH, and red ratio of 8.64 m, 12.16 cm, and 0.4747, with genetic gains of −2.6%, 1.2%, and 8.5%, respectively. Notably, one tree showed marked improvement in all three traits, with genetic gains of 13.1%, 10.1%, and 8.3%.

## 4. Discussion

Understanding the genetic basis and variation in both growth and leaf color traits is crucial for the effective improvement and utilization of *S. discolor*. Through comprehensive field trials and genetic analyses, this study elucidates the main patterns of inheritance and differentiation for these key traits. The following sections provide a detailed discussion of the results for growth characteristics and leaf coloration, and their implications for breeding strategies.

### 4.1. Growth Traits

*S. discolor* demonstrated rapid early growth after plantation establishment. In the second year, mean tree height increased from 1.86 m to 4.10 m, and mean ground diameter from 4.03 cm to 8.06 cm. By the sixth year, mean tree height and DBH reached 7.98 m and 9.99 cm, respectively, but the growth rate slowed in subsequent years. This pattern of rapid initial growth followed by deceleration is consistent with observations in other fast-growing tree species, such as *Populus* and *Eucalyptus* [16,17], and suggests that early selection may be effective in breeding programs.

Analysis of variance indicated that family differences for tree height were not significant in 2015 but became significant or highly significant from 2016 onwards. For DBH, highly significant family differences emerged in 2023. The genetic coefficients of variation among families for tree height in 2020 and 2023, and for DBH in 2023 (0.6%, 1.3%, and 0.9%, respectively), reflect real but limited genetic diversity for these traits. This is comparable to findings in other broadleaved species, where low-to-moderate genetic variation is often reported for growth traits, particularly in advanced generations or improved populations [18,19].

The low-broad-sense heritability for tree height in 2020, and moderate-to-high heritability for tree height and DBH in 2023, indicate that genetic control over these traits increases with stand age—a phenomenon also reported in *Betula platyphylla* and *Liquidambar formosana* [20,21,22]. However, the predominance of non-additive genetic effects, as shown by the low ratio of narrow-sense to broad-sense heritability, suggests that clonal propagation and selection of superior individuals can better capture genetic gains than conventional family or individual selection [13]. This aligns with the strategy recommended for other tree species with strong non-additive effects, such as *Eucalyptus globulus*, *Acacia mangium* and hybrid poplars [23,24,25].

Using the 3σ principle and a 0.5% selection intensity, five clonal candidates were identified for tree height and DBH in 2023. The average tree height, DBH, and red ratio in leaf color of clones selected for tree height were 15.32 m, 18.02 cm, and 0.3558, with genetic gains of 25.2%, 10.8%, and 1.1%, respectively. These results confirm that substantial improvement in growth traits can be achieved through clonal selection, as also demonstrated in *Eucalyptus* breeding programs [26].

### 4.2. Leaf Color Traits

Variance analysis indicated significant family differences in the red ratio of leaf color in 2024, with a genetic coefficient of variation of 0.5% and a broad-sense heritability of 0.21. The narrow-sense heritability was low, and the ratio of narrow-sense to broad-sense heritability was also low (0.06), implying that non-additive genetic effects play a dominant role in the inheritance of this trait. This observation is consistent with studies on leaf color traits in other ornamental or multipurpose trees, where complex inheritance and significant environmental effects are often reported [7,8,9,10,11]. Therefore, as in the case of growth traits, selection of superior clones for vegetative propagation is recommended to maximize genetic gains in leaf color [7,8,27].

Among the five superior clones selected for red leaf ratio, the mean tree height, DBH, and red ratio were 8.64 m, 12.16 cm, and 0.47, with genetic gains of −2.6%, 1.2%, and 8.5%, respectively. Notably, one clone exhibited simultaneous improvement in all three traits, with genetic gains of 13.1%, 10.1%, and 8.3% (Table 8). This highlights the potential for integrated improvement of multiple traits through clonal selection, a strategy supported by recent advances in genomics-assisted breeding [28].

Moreover, highly significant site effects were observed for leaf color, with the mean red ratio in leaf color increasing from south to north (0.31 in Nanjing County, 0.35 in Jiangle County, and 0.36 in Shunchang County). This geographic trend may be related to climatic gradients affecting anthocyanin synthesis and accumulation [29,30,31]. Environmental modulation of coloration is well recognized, and genotype × environment interactions should be taken into account in future breeding and deployment strategies [32,33,34,35].

In summary, both growth and leaf color traits in *S. discolor* are predominantly controlled by non-additive genetic effects, emphasizing the importance of clonal selection for rapid genetic improvement. Early selection based on growth traits is feasible, and integrating site-specific responses will further enhance breeding efficiency.

## 5. Concluding Remarks

Overall, our study demonstrated that both growth and leaf color traits in *S. discolor* were predominantly governed by non-additive genetic effects, with significant influences from environmental variation. For the 10 open-pollinated families evaluated at the three sites in Fujian Province, these results suggest that clonal selection and early trait assessment can be useful for simultaneously improving productivity and ornamental quality. Moreover, the geographic patterns in leaf coloration observed in this trial indicate that genotype × environment interactions should be considered in selection and deployment decisions. Given the relatively limited geographic range and genetic base of the material used here, these conclusions should be interpreted accordingly. Importantly, because Fujian represents only part of the natural distribution range of *S. discolor*, heritability estimates and selection conclusions derived from this study may not be directly transferable to other regions. Given this, we acknowledge that conducting heritability studies across additional distribution regions is therefore necessary to quantify the stability of genetic control across regions (e.g., other provinces in southern China) and to better characterize genotype × environment interactions, thereby enabling region-specific selection and more reliable deployment of improved material. Moving forward, the integration of molecular markers and additional families and broader multi-environment testing would help to further clarify inheritance patterns and to support future genetic improvement and development of *S. discolor* for ecological, economic, and landscape applications.

## Figures and Tables

**Table 1:** Superior tree traits of the tested Families.

No.	Family	Superior Tree Traits		
		Tree Height (m)	DBH (cm)	Clear Bole Height (m)
1	NP01	17.5	17.8	2
2	NP02	15.0	17.6	1.9
3	NP03	20.0	15.0	1.8
4	NP04	16.0	20.8	2.2
5	NP05	20.0	20.0	1.9
6	NP06	15.5	25.0	2.7
7	NJ01	17.5	13.2	2.2
8	NJ02	18.0	17.3	2.3
9	JX01	15.0	16.4	2.8
10	JX02	15.0	14.3	2.1

DBH = Diameter at breast height. Clear bole height = Height to first major branch (the length of the stem without branches). “Family” refers to the number/code of the tested family (provenance or genetic line).

**Table 2:** Phenotypic variation in growth and leaf color traits across families.

Trait	Year	Minimum	Maximum	Mean	Standard Deviation	Coefficient of Variation (%)
Tree height (m)	2015	2.21	2.30	2.26	0.0307	1.4
	2016	3.97	4.26	4.10	0.0933	2.3
	2020	7.56	8.55	7.98	0.2629	3.3
	2023	8.72	9.71	9.25	0.2717	2.9
Basal diameter (cm)	2015	3.90	4.20	4.03	0.1052	2.6
	2016	7.81	8.44	8.06	0.2164	2.7
Diameter at breast height (cm)	2020	9.56	11.03	9.99	0.4217	4.2
	2023	10.99	12.38	11.46	0.3838	3.4
The proportion of red in leaf color	2024	0.3334	0.3423	0.3378	0.0035	1.0

**Table 3:** Analysis of variance for growth and leaf color traits.

Source of Variation	Mean Square								
	2015H	2016H	2020H	2023H	2015D	2016D	2020D	2023D	2024R
Site	32.244 **	366.460 **	2134.228 **	2341.596 **	170.263 **	1013.171 **	3529.587 **	3469.278 **	0.26428 **
Replication	0.919 **	2.765 **	7.277 **	14.396 **	2.234	7.963 *	8.452	13.728 **	0.00296 **
Family	0.189	0.803 *	2.712 *	4.471 **	0.617	4.087	7.521	10.735 *	0.00111 *
Site × Family	0.533 **	1.202 **	2.423 *	2.818 *	2.319 *	9.511 **	9.042 *	8.984 *	0.00103 *
Site × Rep × Family	0.345 **	0.3714 **	3.454 **	3.607 **	1.512	4.397 *	7.233 **	8.674 **	0.00128 **
Error	0.262	0.378	1.339	1.596	1.313	3.504	4.798	5.150	0.00055

H denotes Tree height, D denotes Diameter (at basal or at breast height), R denotes the proportion of red in leaf color, Rep denotes replication. Significance level: * *p* < 0.05, ** *p* < 0.01; otherwise, *p* > 0.05, i.e., not significant. “Family” refers to the number/code of the tested family (provenance or genetic line).

**Table 4:** Variance components and genetic parameters growth and leaf color traits.

Trait	Variance Components				Genetic Parameters			
	Family	Site × Family	Site × Rep × Family	Error	Genetic Coefficient of Variation (%)	$\text{Broad-Sense Heritability}\;h_{f}^{2}$	$\text{Narrow-Sense Heritability}\;h_{i}^{2}$	$h_{i}^{2}$ $/h_{f}^{2}$
2016H	≈0.0000	0.01149	0.07797	0.37837	-	-	-	-
2020H	0.00192	≈0.0000	0.47095	1.33892	0.6	0.0828	0.0042	0.0512
2023H	0.01529	≈0.0000	0.53507	1.60783	1.3	0.3839	0.0283	0.0738
2023D	0.01139	0.02862	0.82883	5.1801	0.9	0.1879	0.0076	0.0403
2024R	0.00000241	≈0.0000	0.00020156	0.00056073	0.5	0.2102	0.0126	0.0600

**Table 5:** Superior trees of clonal progenitors.

Trait	Superior Tree No.	Family	Tree Height in 2023			Diameter at Breast Height in 2023			2024R		
			Mean (m)	Genotypic Value (m)	Genetic Gain (%)	Mean (cm)	Genotypic Value (cm)	Genetic Gain (%)	Mean	Genotypic Value	Genetic Gain (%)
2023H	10	JX02	9.71	0.1753	1.9	12.38	0.1752	1.5	0.3334	−0.000897	−0.3
2023D	10	JX02	9.71	0.1753	1.9	12.38	0.1752	1.5	0.3334	−0.000897	−0.3
2024R	4	NP04	9.45	0.0777	0.8	11.27	−0.0340	−0.3	0.3423	0.000972	0.3
All traits	8	NJ02	9.40	0.0590	0.6	11.53	0.0151	0.1	0.3419	0.000881	0.3
Population Mean			9.25	-	-	11.46	-	-	0.3377	-	-

“Genotypic Value” refers to the estimated genotypic (breeding) value for the trait. “Genetic Gain (%)” indicates the percent increase (or decrease) compared to the population mean. “All traits” refers to the superior tree selected based on both growth and leaf color traits. Population mean is provided for reference.

**Table 6:** Clonal progenitors selected based on tree height in 2023.

Site	Replication	Family	Tree No.	Tree Height in 2023			Diameter at Breast Height in 2023			2024R		
				Mean (m)	Genotypic Value (m)	Genetic Gain (%)	Mean (cm)	Genotypic Value (cm)	Genetic Gain (%)	Mean	Genotypic Value	Genetic Gain (%)
1	2	4	8	16.30	2.7065	29.3	22.20	2.0199	17.6	0.3698	0.006747	2.0
1	7	8	4	15.40	2.3610	25.5	16.80	1.0053	8.8	0.3950	0.012044	3.6
1	10	4	3	15.20	2.2842	24.7	19.90	1.5878	13.9	0.3403	0.000547	0.2
3	5	4	3	15.00	2.2074	23.9	10.20	−0.2349	−2.1	0.2885	−0.010342	−3.1
1	8	7	7	14.70	2.0923	22.6	21.00	1.7944	15.7	0.3854	0.010027	3.0
Mean				15.32	2.3303	25.2	18.02	1.2345	10.8	0.3558	0.003805	1.1
Population Mean				9.25	-	-	11.46	-	-	0.3377	-	-

**Table 7:** Clonal progenitors selected based on diameter at breast height in 2023.

Site	Replication	Family	Tree No.	Tree Height in 2023			DBH in 2023			2024R		
				Mean (m)	Genotypic Value (m)	Genetic Gain (%)	Mean (cm)	Genotypic Value (cm)	Genetic Gain (%)	Mean	Genotypic Value	Genetic Gain (%)
3	10	6	3	10.50	0.4799	5.2	23.0	2.1702	19.0	0.3036	−0.007168	−2.1
1	2	4	8	16.30	2.7065	29.3	22.2	2.0199	17.6	0.3698	0.006747	2.0
1	8	7	7	14.70	2.0923	22.6	21.0	1.7944	15.7	0.3854	0.010027	3.0
3	5	5	3	13.00	1.4396	15.6	21.0	1.7944	15.7	0.2946	−0.009060	−2.7
1	9	10	1	11.60	0.9022	9.8	20.8	1.7569	15.3	0.3451	0.001555	0.5
Mean				13.22	1.5241	16.5	21.6	1.9072	16.7	0.3397	0.000420	0.1
Population Mean				9.25	-	-	11.5	-	-	0.3377	-	-

**Table 8:** Clonal progenitors selected based on the proportion of red in leaf color in 2024.

Site	Replication	Family	Tree No.	Tree Height in 2023			DBH in 2023			2024R		
				Mean (m)	Genotypic Value (m)	Genetic Gain (%)	Mean (cm)	Genotypic Value (cm)	Genetic Gain (%)	Mean	Genotypic Value	Genetic Gain (%)
2	7	6	2	6.69	−0.9828	−10.6	6.60	−0.9113	−8.0	0.5667	0.048136	14.3
1	4	7	8	12.40	1.2093	13.1	17.60	1.1556	10.1	0.4710	0.028020	8.3
1	3	10	2	11.00	0.6718	7.3	16.30	0.9113	8.0	0.4562	0.024909	7.4
1	1	2	1	8.00	−0.4799	−5.2	14.80	0.6295	5.5	0.4401	0.021524	6.4
2	6	1	2	5.09	−1.5970	−17.3	5.50	−1.1180	−10.0	0.4395	0.021398	6.3
Mean				8.64	−0.2357	−2.6	12.16	0.1334	1.2	0.4747	0.028797	8.5
Population Mean				9.25	-	-	11.46	-	-	0.3377	-	-

## Data Availability

Data are contained within the article.
